# Case report: Heterogenous SMARCA4-deficient thoracic non-small cell lung carcinoma with various responses to nivolumab

**DOI:** 10.3389/fimmu.2023.1131448

**Published:** 2023-03-27

**Authors:** Yun-Tzu Lin, Chien-Feng Li, Hung-Chang Wu, Yi-Hua Jan, Yu-Hsuan Kuo

**Affiliations:** ^1^ Department of Oncology, Chi-Mei Medical Center, Tainan, Taiwan; ^2^ Department of Pathology, Chi-Mei Medical Center, Tainan, Taiwan; ^3^ Institute of Precision Medicine, National Sun Yat-sen University, Kaohsiung, Taiwan; ^4^ Department of Medical Research, Chi Mei Medical Center, Tainan, Taiwan; ^5^ National Institute of Cancer Research, National Health Research Institutes, Tainan, Taiwan; ^6^ College of Pharmacy and Science, Chia Nan University, Tainan, Taiwan; ^7^ ACT Genomics Co. Ltd., Taipei, Taiwan; ^8^ Institute of Biomedical Sciences, National Sun Yat-sen University, Kaohsiung, Taiwan

**Keywords:** SMARCA4, BRG1, SWI/SNF, lung cancer, non-small cell lung carcinoma, check-point inhibitors, nivolumab

## Abstract

SMARCA4-deficient non-small cell carcinoma is an aggressive neoplasm with poor outcome. Several studies have highlighted its immunochemistry, pathophysiology, and underlying mechanisms, but studies of its definite treatment are few. Here, we report on a 69-year-old male with heterogenous pathological presentations of SMARCA4-deficient non-small cell carcinoma. He initially presented with neck lymphadenopathies. Immunohistochemistry staining and genomic profiling confirmed the diagnosis of SMARCA4-deficient non-small cell carcinoma. The patient responded well to immune checkpoint inhibitors with nivolumab. However, new lesions with various pathological presentations and various responses to nivolumab appeared during the treatment course. The patient survived more than 3 years from the initial diagnosis. This case shows the efficacy of nivolumab to treat SMARCA4-deficient non-small cell lung carcinoma.

## 1 Introduction

SMARCA4 (BRG1), a central component of the SWI/SNF chromatin remodeling complex, influences transcriptional regulation by disrupting the histone-DNA contacts in an ATP-dependent manner (1). Inactivating mutations and loss of expression of this complex have been implicated in the carcinogenesis of several cancers such as small cell carcinoma of the ovary hypercalcemic type (SCCOHT), medulloblastoma, lung cancer, and pancreatic cancer (2–8). SMARCA4 is generally believed to be a tumor suppressor gene in primary lung tumors, and its regulation of gene expression is essential for growth arrest and cell senescence; hence, it is a critical entity in cancer progression (8–10).

Recently, the 5^th^ edition of the WHO classification of thoracic tumors classified this entity into SMARCA-4 deficient undifferentiated thoracic tumors (SMARCA-4 DUT) and SMARCA4-deficient non-small cell lung carcinomas (SMARCA4-dNSCLC). Both are associated with smoking and male preponderance. In non-small cell lung carcinomas (NSCLCs), we usually assess molecular markers to determine our clinical practice. However, SMARCA4-dNSCLC lacks alterations in currently targetable oncogenic drivers, such as epidermal growth factor receptor (EGFR), anaplastic lymphoma kinase (ALK), and c-ros oncogene 1 (ROS1) (11). Currently, no standard treatment exists for SMARCA4-dNSCLC. We present the case of a patient with heterogenous SMARCA4-dNSCLC with various responses to nivolumab.

## 2 Case description

**Figure 1:**
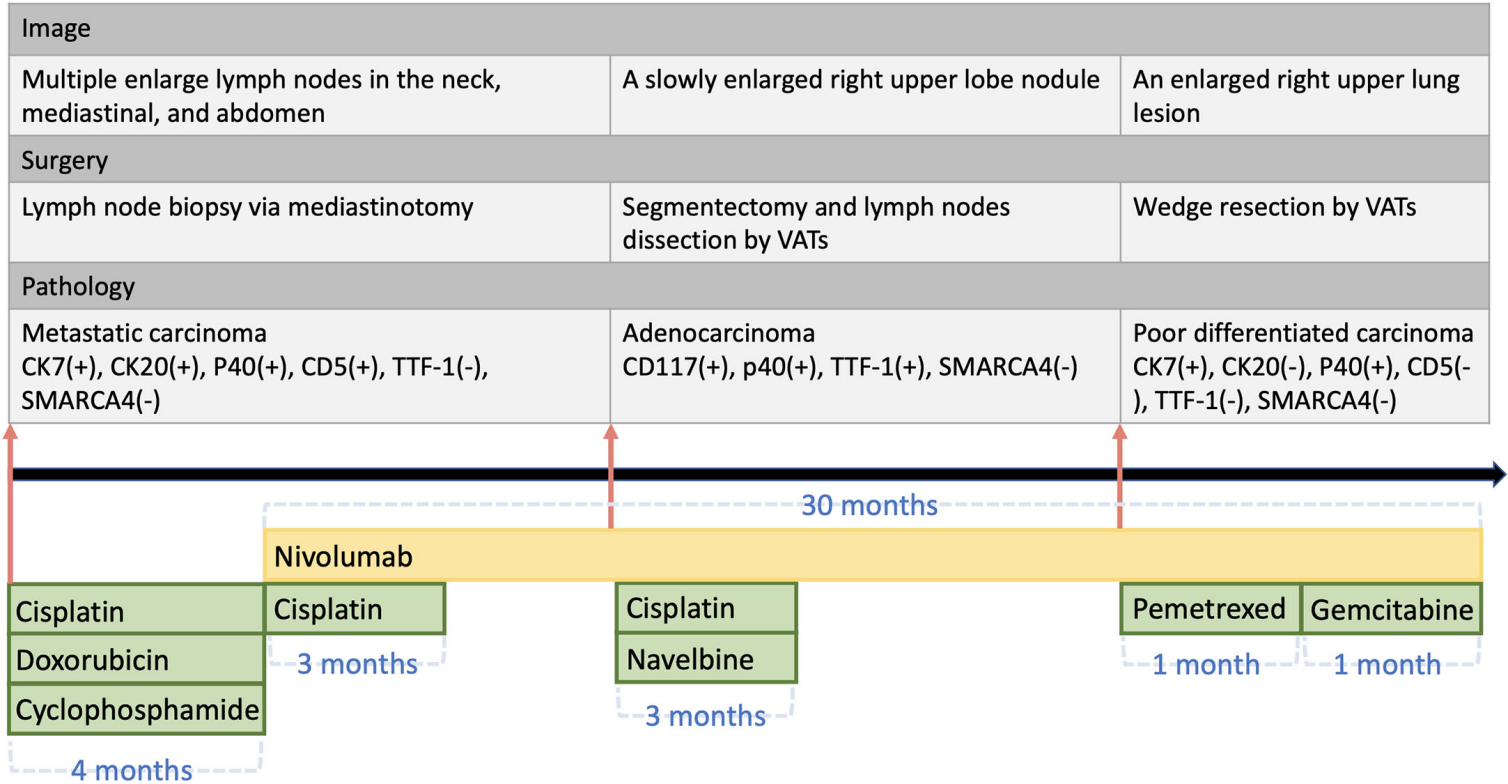
Timeline of the clinical course of the patient.

**Figure 2:**
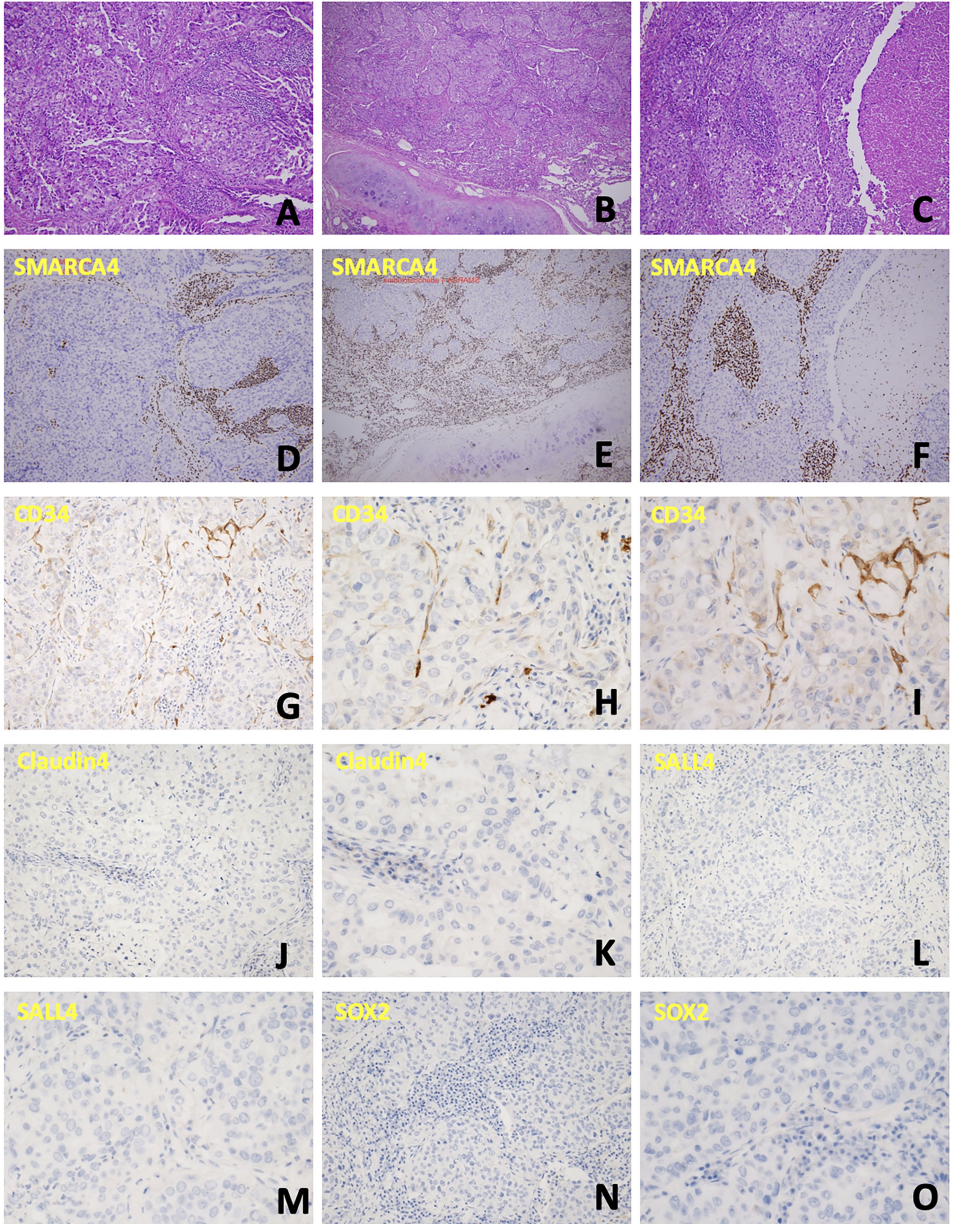
Cellular characterization of the disease course. **(A)** Neoplastic cells arranged in confluent papillary and glandular structures. **(B)** The tumor was arranged in infiltrative nests, with necrosis and focal extracellular and intracellular mucin production noted. **(C)** Tumor cells were arranged in solid nests and occasional papillary structure, with lymphovascular invasion also present. **(D-F)** Immunochemical staining to detect SMARCA4 corresponding to the above specimen; all these tumors cells were negative for SMARCA4 expression. **(G-I)** CD34 is weakly positive. **(J, K)** Claudin4 is negative. **(L, M)** SALL4 is negative. **(N, O)** SOX2 is negative.

The patient’s clinical course is illustrated in **Figure 1**. A 69-year-old man initially presented with neck lymphadenopathies. Chest computed tomography revealed multiple enlarged lymph nodes in the neck, mediastinal mass, and abdomen. Right upper lobe and left upper lobe lung nodules were also noted. Lymph node biopsy *via* mediastinotomy was performed and pathology demonstrated metastatic carcinoma (**Figure 2A**). Tumor cells were immuno-active for CK7; focally positive for CK20, P40, CD34 and CD5; but negative for TTF-1, Claudin4, SALL4, SOX2 and SMARCA4 (**Figure 2**). Tumor cells also had low PD-L1 expression (PD-L1 22C3 immunohistochemistry Combined Positive Score < 1). To identify actionable mutations, the tumor biopsy specimen was sequenced using ACTOnco^®^, a comprehensive genomic panel (CGP) of 440 cancer-related genes. Genomic profiling showed a high tumor mutational burden (TMB, 16.9 muts/Mb) with 40 nonsynonymous mutations identified. Among them, biallelic loss-of-function mutation in SMARCA4 along with 4 other mutations (BARD1, ERBB3, MED12, and TP53) were considered clinically relevant variants (**Table 1**). Furthermore, an amplified genomic region encoding the INPP4B gene at chromosome 4 was identified, with a copy number of 13. Eleven genes with heterozygous deletions (FBXW7, RAD50, CDKN2A, PTCH1, TSC1, FLCN, TP53, PALB2, SMAD4, SMARCA4, and STK11) were also identified. The patient underwent six cycles of cyclophosphamide, doxorubicin, and cisplatin treatment. His tumor regressed after treatment. We switched the regimen to cisplatin 40 mg/m2 combined with nivolumab 140 mg every 3 weeks for six cycles. Chest computed tomography showed further regression of the main tumor, in the mediastinal mass, and in the neck lymph nodes. Then, we changed the regimen to nivolumab monotherapy 140 mg every 3 weeks for three more months. However, chest computed tomography revealed a slowly enlarged right upper lobe nodule while other lesions remained stable. The patient underwent segmentectomy for enlarged right upper lobe nodule and lymph node dissection by Video-Assisted Thoracoscopic Surgery. Pathology disclosed adenocarcinoma (**Figure 2C**) this time. The tumor cells were positive for TTF-1; weakly positive for CD117 and p40; but still negative for SMARCA4 (**Figure 2D**). The patient underwent chemotherapy with four cycles of vinorelbine and cisplatin. Meanwhile, nivolumab 140 mg every 3 weeks was maintained for a further 20 months. However, chest computed tomography showed an enlarged right upper lung lesion while other lesions were stable. The patient underwent another wedge resection by video-assisted thoracoscopic surgery. Pathology this time disclosed poorly differentiated carcinoma (**Figure 2E**). Immunohistochemically, the tumor cells were positive for CK7; occasionally positive for p40; but not positive for TTF-1, CK20, CD5, and SMARCA4 (**Figure 2F**). However, the tumor progressed rapidly this time, and the patient’s condition deteriorated, even though we changed the chemotherapy regimen to nivolumab plus pemetrexed, and subsequently nivolumab plus gemcitabine. The patient survived for 37 months from initial diagnosis.

**Table 1:** Next-generation sequencing of detected gene mutations in the tumor biopsy specimen.

Gene	Chr	Exon	Amino Acid Change	Coverage	Allele Frequency
*BARD1*	2	–	Splice acceptor	1071	24.5%
*ERBB3*	12	3	p.V104L	2276	24.8%
*MED12*	X	–	Splice donor	650	47.7%
*SMARCA4*	19	29	p.E1310*	1428	31.9%
*TP53*	17	5	p.V157F	1008	33.9%

"*" means stop-gain, and "-" means the mutation occurs in intron but not exon.

## 3 Discussion

SMARCA4-deficient thoracic tumors are divided into 2 types: SMARCA4-deficient sarcoma and SMARCA4-dNSCLC (12). The 5^th^ edition of the WHO classification of thoracic tumors recently classified this entity into SMARCA-4 DUT and SMARCA4-dNSCLC. SMARCA-4 DUT always arises outside the lung parenchyma, commonly at the mediastinum, and involvement of adjacent structures such as airway, esophagus, or thymus is mentioned frequently. It could also present at the lung parenchyma, pulmonary hilum, or pleura with or without chest wall invasion, and it is highly indicative of peritoneal metastases (11). SMARCA4-dNSCLC is mainly intrapulmonary and presents with invasion to the pleura and vascular structures (13, 14). Both SMARCA-4 DUT and SMARCA4-dNSCLC are associated with smoking and male preponderance. Morphological overlaps are seen between these two entities; however, the exact link between the two is still being studied.

As the poorly differentiated and rhabdoid morphology of SMARCA4-dNSCLC necessitates consideration of a broad spectrum of differential diagnoses, a comprehensive IHC panel is frequently required to aid in the diagnosis of SMARCA4-dNSCLC. The differential diagnosis would include neuroendocrine carcinoma, large cell carcinoma, lymphomas, NUT carcinoma, melanomas, and various other sarcomas (9). Furthermore, we can differentiate SMARCA4-dNSCLC from SMARCA-4 DUT by several immunohistochemical stains such as CD34, Claudin4, SOX2, and SALL4 which are usually strongly positive in SMARCA-4 DUT, but not in SMARCA4-dNSCLC (9, 11). In our case, although CD34 had weak positive in staining, Claudin4, SOX2 and SALL4 were all negative in staining which confirmed the diagnosis of SMARCA-4 dNSCLC.

SMARCA4-dNSCLC is a new disease entity with an aggressive clinical course and poor prognosis. The characteristics of these patients include male, younger age, and being a smoker. The reported median survival is 6 months, and the standard treatment has not yet been established (15). SMARCA4-dNSCLC tumors have a uniform lack of the actionable gene alterations found in conventional lung adenocarcinomas; namely, EGFR mutation, EML4-ALK rearrangement, and ROS1 rearrangement (14). In preclinical models of SMARCA4-deficient tumors, CDK4/6, ATR, AURKA, and EZH2 inhibition showed antitumor activity (16). Several cases have shown that SMARCA4-dNSCLC is chemo-sensitive to platinum-based chemotherapy (14). Low expression of SMARCA4/BRG1 is a predictive biomarker for increased sensitivity to platinum-based chemotherapy in NSCLC. The possible mechanism is that the defects in DNA repair result in cisplatin sensitivity in lung cancers with BRG1 knockdown, so further studies using platinum-based chemotherapy and other therapies targeting DNA repair are needed (17).

Recently, several case reports showed that using an immune checkpoint inhibitor was effective in treating SMARCA4-deficient thoracic tumors. We summarized various treatments and its effect on SMARCA4-dNSCLC and SMARCA4-DUT in **Table 2**. Naito et al. reported on a 43-year-old male patient with SMARCA4-dNSCLC who received nivolumab as the fourth-line treatment. Whole exome sequencing revealed a high TMB despite lack of PD-L1 expression. Disease control was maintained for more than 14 months after continuous tumor shrinkage through 22 doses of nivolumab (18). Henon et al. reported on a patient with a SMARCA4-deficient malignant rhabdoid-like tumor who had a long-lasting response with pembrolizumab. Partial response (72%) was achieved after 11 months of treatment, although the TMB was remarkably low, and the immunohistochemistry stain was negative for PD-L1 (23). In another case, an initially unresectable SMARCA4-deficient thoracic non-small cell carcinoma was treated using nivolumab, then successfully resected, followed by adjuvant chemotherapy (13). Recently, a biomarkers analysis from CHOICE-01, a phase 3 study of toripalimab versus placebo in combination with first-line chemotherapy for advanced NSCLC without EGFR/ALK mutations, revealed that patients in the toripalimab arm harboring the SMARCA4 mutation achieved significantly better progression-free survival (26). SMARCA4-deficient tumors have been found to have high TMB, mainly a biallelic inactivation by nonsense and frameshift mutational tumorigenesis, less commonly missense mutation, splice-site mutation, deletion with loss of heterozygosity or deletion alone, or with a second mutation (27). For our patient, the tumor cells showed low PD-L1 expression but high TMB. The high TMB may explain his long duration of response to immunotherapy. Interestingly, for our patient, the next-generation sequence showed heterozygous deletion of STK11, which was shown to be associated with resistance to immune checkpoint inhibitors. However, SMARCA4-mutated NSCLCs have been reported to frequently harbor coexisting mutations in TP53, KRAS, CDKN2A, and STK11 (28–31). The coexisting mutations in SMARCA4 and STK11 seem to not influence the treatment effect of checkpoint inhibitors in our case.

**Table 2:** Comparison the effect of various treatments on SMARCA4-dNSCLC and SMARCA-4 DUT).

Reference	Age/sex	Smoking	TNM	PD-L1	Therapy	Outcome
SMARCA4-deficient non small cell lung carcinoma (SMARCA4-dNSCLC)						
Naito T, et al. (18)	43/M	Smoker	pT4N0M0	0%	1st: carboplatin (AUC 5–6, day 1), paclitaxel (180-200 mg/m2, day 1), and bevacizumab (15 mg/kg, day 1)	SD
					2nd: one cycles of docetaxel (50-60 mg/m2, day 1) and ramucirumab (10 mg/kg, day 1)	SD
					3rd: two cycles of pemetrexed (500 mg/m2, day 1)	PD
					4th: Nivolumab 3mg/kg, Q2W	PR for more than 14 months
Nambirajan A, et al. (19)	67/M	Unknown	II	Unknown	Left upper lobectomy without adjuvant therapy	Alive without disease (12months)
SMARCA4-deficient underentiated tumor (SMARCA4-DUT)						
Takada K, et al. (20)	70/F	Unknown	IV	>60%	Pembrolizumab for 8 cycles	PR after one dose of Pembrolizumab
Kawachi H, et al. (21)	73/F	Current smoker (53 pack-years)	IVB	40%	Atezolizumab, bevacizumab, paclitaxel, and carboplatin (ABCP) for 3 cycles then Atezolizumab, bevacizumab (AB) maintenance	PR after 2 courses of ABCP No PD for 17 months
	59/M	Current smoker (39 pack-years)	IVB	0%	ABCP for 3 cycles then AB maintenance	PR after 2 courses of ABCP
	64/F	Past smoker (44 pack-years)	IVB	80%	ABCP for 3 cycles then AB maintenance	PR after 3 cycles of ABCP
Shi L, et al. (22)	50/M	Current smoker (36 pack-years)	Unknown	90%	Tislelizumab for 6 cycles	PR after 6 cycles of Tislelizumab
Henon C, at al (23).	58/F	Unknown	IV	0%	1st: 12 Gy in three fractions decompressive mediastinal radiation therapy and a first-line chemotherapy including carboplatin and weekly paclitaxel	PD
					2nd: Pembrolizumab 200mg Q3W	PR for 11 months
Kunimasa K, et al. (24)	51/M	Current smoker (22.5 pack-years)	IVA	0%	ABCP for 6 cycles	PR (converse to surgery) No recurrence for 9 months
Utsumi T, et al. (25)	72/M	Smoker (80 pack-years)	Unknown	Unknown	Atezolizumab, carboplatin, nab-paclitaxel	SD for 7 months

Complete loss of SMARCA4 was observed in 5.5% of evaluable pulmonary adenocarcinomas or squamous cell carcinomas. Of those adenocarcinomas with loss of SMARCA4, 80% were TTF1 negative (32). In our case, the tumor morphology was obviously different from SMARCA4-deficient thoracic sarcoma, although the lung tumor cells stained differently for TTF-1, which confirmed the diagnosis of SMARCA4-deficient thoracic NSCLC. Our patient had several lung lesions, some TTF-1 positive and some TTF-1 negative. The initially TTF-1 negative lesions had good response to nivolumab treatment. However, resistance developed, and second lesions appeared which were TTF-1 positive. The last refractory lesion turned TTF-1 negative again. It appears that although the immunohistochemical staining was SMARCA-4 negative throughout, the TTF-1 staining varied from time to time. The relationship between TTF-1 immunostaining and treatment response of check point inhibitors is not yet clear and likely warrants further investigation.

In conclusion, the diagnosis of SMARCA-deficient thoracic NSCLC should be kept in mind for patients with poorly differentiated lung carcinoma or histologically atypical lung cancer. Next generation sequence might also be helpful in treatment planning. Currently, no established treatment exists for SMARCA4-deficient thoracic carcinoma. We present a case with SMARCA4-dNSCLC who had prolonged response to nivolumab. A larger study of SMARCA4-dNSCLC is needed to validate the efficacy of checkpoint inhibitors.

## Data availability statement

The original contributions presented in the study are included in the article/supplementary materials, further inquiries can be directed to the corresponding author/s.

## Ethics statement

The studies involving human participants were reviewed and approved by the ethics committee of Chi Mei Medical Center. The ethics committee waived the requirement of written informed consent for the publication of any identifiable data/information.

## Author contributions

Y-TL: wrote the manuscript and searched the literature. C-FL: provided histopathology and next-generation sequencing (NGS) analysis. H-CW: offered the case and treated the patient. Y-HJ: provided NGS analysis. Y-HK: offered the case, treated the patient, wrote the manuscript, searched the literature, and revised the manuscript. All authors contributed to the article and approved the submitted version.

## References

[1] Roberts CW Orkin SH . The SWI/SNF complex–chromatin and cancer. Nat Rev Cancer (2004) 4(2):133–42. doi: 10.1038/nrc1273 14964309

[2] Witkowski L Carrot-Zhang J Albrecht S Fahiminiya S Hamel N Tomiak E . Germline and somatic SMARCA4 mutations characterize small cell carcinoma of the ovary, hypercalcemic type. Nat Genet (2014) 46(5):438–43. doi: 10.1038/ng.2931 24658002

[3] Ramos P Karnezis AN Hendricks WP Wang Y Tembe W Zismann VL . Loss of the tumor suppressor SMARCA4 in small cell carcinoma of the ovary, hypercalcemic type (SCCOHT). Rare Dis (2014) 2(1):e967148. doi: 10.4161/2167549X.2014.967148 26942101 PMC4755243

[4] Jelinic P Mueller JJ Olvera N Dao F Scott SN Shah R . Recurrent SMARCA4 mutations in small cell carcinoma of the ovary. Nat Genet (2014) 46(5):424–6. doi: 10.1038/ng.2922 PMC5699446 24658004

[5] Parsons DW Li M Zhang X Jones S Leary RJ Lin JC . The genetic landscape of the childhood cancer medulloblastoma. Science (2011) 331(6016):435–9. doi: 10.1126/science.1198056 PMC3110744 21163964

[6] Medina PP Romero OA Kohno T Montuenga LM Pio R Yokota J . Frequent BRG1/SMARCA4-inactivating mutations in human lung cancer cell lines. Hum Mutat (2008) 29(5):617–22. doi: 10.1002/humu.20730 18386774

[7] Reisman DN Sciarrotta J Wang W Funkhouser WK Weissman BE . Loss of BRG1/BRM in human lung cancer cell lines and primary lung cancers: correlation with poor prognosis. Cancer Res (2003) 63(3):560–6. 12566296

[8] Wong AK Shanahan F Chen Y Lian L Ha P Hendricks K . BRG1, a component of the SWI-SNF complex, is mutated in multiple human tumor cell lines. Cancer Res (2000) 60(21):6171–7. 11085541

[9] Chatzopoulos K Boland JM . Update on genetically defined lung neoplasms: NUT carcinoma and thoracic SMARCA4-deficient undifferentiated tumors. Virchows Arch (2021) 478(1):21–30. doi: 10.1007/s00428-020-03011-3 33409598

[10] Medina PP Carretero J Fraga MF Esteller M Sidransky D Sanchez-Cespedes M . Genetic and epigenetic screening for gene alterations of the chromatin-remodeling factor, SMARCA4/BRG1, in lung tumors. Genes Chromosomes Cancer (2004) 41(2):170–7. doi: 10.1002/gcc.20068 15287030

[11] Nambirajan A Jain D . Recent updates in thoracic SMARCA4-deficient undifferentiated tumor. Semin Diagn Pathol (2021) 38(5):83–9. doi: 10.1053/j.semdp.2021.06.001 34147303

[12] Decroix E Leroy K Wislez M Fournel L Alifano M Damotte D . [SMARCA4-deficient thoracic tumors: A new entity]. Bull Cancer (2020) 107(1):41–7. doi: 10.1016/j.bulcan.2019.12.001 31916995

[13] Naito T Udagawa H Umemura S Sakai T Zenke Y Kirita K . Non-small cell lung cancer with loss of expression of the SWI/SNF complex is associated with aggressive clinicopathological features, PD-L1-positive status, and high tumor mutation burden. Lung Cancer (2019) 138:35–42. doi: 10.1016/j.lungcan.2019.10.009 31630044

[14] Agaimy A Fuchs F Moskalev EA Sirbu H Hartmann A Haller F . SMARCA4-deficient pulmonary adenocarcinoma: clinicopathological, immunohistochemical, and molecular characteristics of a novel aggressive neoplasm with a consistent TTF1(neg)/CK7(pos)/HepPar-1(pos) immunophenotype. Virchows Arch (2017) 471(5):599–609. doi: 10.1007/s00428-017-2148-5 28555282

[15] Perret R Chalabreysse L Watson S Serre I Garcia S Forest F . SMARCA4-deficient thoracic sarcomas: Clinicopathologic study of 30 cases with an emphasis on their nosology and differential diagnoses. Am J Surg Pathol (2019) 43(4):455–65. doi: 10.1097/PAS.0000000000001188 30451731

[16] Schoenfeld AJ Bandlamudi C Lavery JA Montecalvo J Namakydoust A Rizvi H . The genomic landscape of SMARCA4 alterations and associations with outcomes in patients with lung cancer. Clin Cancer Res (2020) 26(21):5701–8. doi: 10.1158/1078-0432.CCR-20-1825 PMC7641983 32709715

[17] Bell EH Chakraborty AR Mo X Liu Z Shilo K Kirste S . SMARCA4/BRG1 is a novel prognostic biomarker predictive of cisplatin-based chemotherapy outcomes in resected non-small cell lung cancer. Clin Cancer Res (2016) 22(10):2396–404. doi: 10.1158/1078-0432.CCR-15-1468 PMC4867280 26671993

[18] Naito T Umemura S Nakamura H Zenke Y Udagawa H Kirita K . Successful treatment with nivolumab for SMARCA4-deficient non-small cell lung carcinoma with a high tumor mutation burden: A case report. Thorac Cancer (2019) 10(5):1285–8. doi: 10.1111/1759-7714.13070 PMC6501032 30972962

[19] Nambirajan A Singh V Bhardwaj N Mittal S Kumar S Jain D . SMARCA4/BRG1-deficient non-small cell lung carcinomas: A case series and review of the literature. Arch Pathol Lab Med (2021) 145(1):90–8. doi: 10.5858/arpa.2019-0633-OA 33367658

[20] Takada K Sugita S Murase K Kikuchi T Oomori G Ito R . Exceptionally rapid response to pembrolizumab in a SMARCA4-deficient thoracic sarcoma overexpressing PD-L1: A case report. Thorac Cancer (2019) 10(12):2312–5. doi: 10.1111/1759-7714.13215 PMC6885443 31617320

[21] Kawachi H Kunimasa K Kukita Y Nakamura H Honma K Kawamura T . Atezolizumab with bevacizumab, paclitaxel and carboplatin was effective for patients with SMARCA4-deficient thoracic sarcoma. Immunotherapy (2021) 13(10):799–806. doi: 10.2217/imt-2020-0311 34030451

[22] Shi L Lin L Ding Y Zeng Y Chen X . Case report: A rapid response to immunotherapy in a thoracic SMARCA4-deficient undifferentiated tumor with respiratory failure. Front Oncol (2022) 12:1020875. doi: 10.3389/fonc.2022.1020875 36452500 PMC9703460

[23] Henon C Blay JY Massard C Mir O Bahleda R Dumont S . Long lasting major response to pembrolizumab in a thoracic malignant rhabdoid-like SMARCA4-deficient tumor. Ann Oncol (2019) 30(8):1401–3. doi: 10.1093/annonc/mdz160 31114851

[24] Kunimasa K Okami J Takenaka S Honma K Kukita Y Nagata S . Conversion surgery for advanced thoracic SMARCA4-deficient undifferentiated tumor with atezolizumab in combination with bevacizumab, paclitaxel, and carboplatin treatment: A case report. JTO Clin Res Rep (2021) 2(11):100235. doi: 10.1016/j.jtocrr.2021.100235 34746887 PMC8551843

[25] Utsumi T Taniguchi Y Noda Y Fukai M Kibata K Murakawa T . SMARCA4-deficient undifferentiated tumor that responded to chemotherapy in combination with immune checkpoint inhibitors: A case report. Thorac Cancer (2022) 13(15):2264–6. doi: 10.1111/1759-7714.14547 PMC9346173 35778998

[26] Wang Z Wu L Li B Cheng Y Li X Wang X . Toripalimab Plus Chemotherapy for Patients With Treatment-Naive Advanced Non-Small-Cell Lung Cancer: A Multicenter Randomized Phase III Trial (CHOICE-01). J Clin Oncol. 2023;41 (3):651–63. 10.1200/JCO.22.00727 PMC9870236 36206498

[27] Armon S Hofman P Ilié M . Perspectives and issues in the assessment of SMARCA4 deficiency in the management of lung cancer patients. Cells (2021) 10(8):1920. doi: 10.3390/cells10081920 34440689 PMC8394288

[28] Medina PP Carretero J Ballestar E Angulo B Lopez-Rios F Esteller M . Transcriptional targets of the chromatin-remodelling factor SMARCA4/BRG1 in lung cancer cells. Hum Mol Genet (2005) 14(7):973–82. doi: 10.1093/hmg/ddi091 15731117

[29] Liu J Lee W Jiang Z Chen Z Jhunjhunwala S Haverty PM . Genome and transcriptome sequencing of lung cancers reveal diverse mutational and splicing events. Genome Res (2012) 22(12):2315–27. doi: 10.1101/gr.140988.112 PMC3514662 23033341

[30] Seo JS Ju YS Lee WC Shin JY Lee JK Bleazard T . The transcriptional landscape and mutational profile of lung adenocarcinoma. Genome Res (2012) 22(11):2109–19. doi: 10.1101/gr.145144.112 PMC3483540 22975805

[31] Cancer Genome Atlas Research N . Comprehensive molecular profiling of lung adenocarcinoma. Nature (2014) 511(7511):543–50. doi: 10.1038/nature13385 PMC4231481 25079552

[32] Herpel E Rieker RJ Dienemann H Muley T Meister M Hartmann A . SMARCA4 and SMARCA2 deficiency in non-small cell lung cancer: immunohistochemical survey of 316 consecutive specimens. Ann Diagn Pathol (2017) 26:47–51. doi: 10.1016/j.anndiagpath.2016.10.006 28038711

